# Spectral dependence of nonlinear absorption in ordered silver metallic nanoprism arrays

**DOI:** 10.1038/s41598-017-04814-2

**Published:** 2017-07-13

**Authors:** Héctor Sánchez-Esquivel, Karen Y. Raygoza-Sánchez, Raúl Rangel-Rojo, Emanuele Gemo, Niccolò Michieli, Boris Kalinic, Jorge Alejandro Reyes-Esqueda, Tiziana Cesca, Giovanni Mattei

**Affiliations:** 1 Departamento de Óptica, División Física Aplicada, CICESE, A.P. 360, Ensenada, 22860 BC Mexico; 2 Dipartimento di Fisica e Astronomia Galileo Galilei, Università degli Studi di Padova, Padova, Italy; 3 Instituto de Fisica, Universidad Nacional Autónoma de México, Circuito de la Investigación Científica, Ciudad Universitaria, Delegación Coyoacán, C.P. 4510 Ciudad de México, Mexico

## Abstract

Ordered metallic nanoprism arrays have been proposed as novel and versatile systems for the observation of nonlinear effects such as *nonlinear absorption*. The study of the effect of the local field reinforcement on the fast optical third order nonlinear response around the *Surface Plasmon Resonance* is of great interest for many plasmonic applications. In this work, silver nanoprism arrays have been synthesized by the *nanosphere lithography* method. A low repetition rate tunable picosecond laser source was used to study the irradiance and wavelength dependence of the *nonlinear absorption* properties around the dipolar and quadrupolar resonances of the nanoarray with the use of the *z*-*scan technique*. The irradiance dependence of the on-resonance nonlinearity was studied, and a spectral region where nonlinear absorption is negligible was identified. This is important for the possible application of these materials in optical information processing devices.

## Introduction

The promise of novel materials with a-la-carte optical properties has placed nanostructures in the focus of intense research activity for applications such as all-optical switching, biomedics and solar-energy^1–4^. In the case of metal nanoparticles in dielectric media, an effect known as *localized surface plasmon resonance* (LSPR) has been studied for quite some time^5^. These resonances are understood as collective oscillations of the free electrons localized along the metal nanoparticle surface with an associated confined electromagnetic field. As the dimensions of these nanoparticles approach tens of nanometers, the evanescent local polariton wave experiences dielectric confinement, increasing the effective third order electric susceptibility ${\chi }_{eff}^{\mathrm{(3)}}$ of the medium^6–8^. Also, the presence of vertices in the metallic nanoparticle morphology has been shown to further increase the enhancement of the nonlinear properties of the composite material^9^. Because of this, nanoparticle design shifted quickly towards geometries featuring a greater number and curvature of their vertices. Also when two or more nanoparticles are present in close proximity, such configurations are sometimes called nano-antennas, providing parallelism with the much more known electrical devices, resulting in enhanced electric field amplitudes^10^ and therefore, in an enhanced effective nonlinear response as well. Furthermore, as the number of ordered and close-proximity nanoparticles is increased, the total electric field reinforcement of the ensemble approaches the result of the coherent sum of their elementary contributions. As such, it has been demonstrated that arrays of ordered nanoparticles offer greater nonlinear response than their disordered counterparts^11^. One nanoparticle geometry that has gained popularity in recent years is the plasmonic nanoprism^12^, and more specifically, *metallic nanoprism arrays* (MNPAs) embedded in dielectrics. Structures such as MNPAs take advantage of all the previously mentioned nonlinear enhancement properties and thus are positioned as attractive potential hosts for applications based on third-order nonlinear effects such as *nonlinear absorption* (NLA) and *nonlinear refraction* (NLR)^13^. Amongst the wide gamut of techniques for nanoparticle manufacturing, great weight is placed on fabrication time, cost, and difficulty. In these terms, *nanosphere lithography* (NSL) is an attractive technique for the production of MNPAs. With this technique, greater control of the material LSPR properties is gained by simple modification of the fabrication parameters^12^.

In this work we provide a study of the rich NLA behavior of MNPAs fabricated by NSL, using the single beam z-scan technique^14^, which allows to determine the magnitude and sign of the NLA effects. A tunable laser source with pulse duration in the picosecond range and low pulse repetition rate was used to study the NLA around the dipolar resonance LSPR features of the nanoarray. This scheme is of special interest due to its ability to observe fast responses as well as rendering negligible thermal contribution. A study of the dependence of the NLA of MNPAs as a function of the input irradiance and the peak absorption wavelength is also presented.

## Experimental Section

### Synthesis of ordered metallic nanoprism arrays

**Figure 1:**
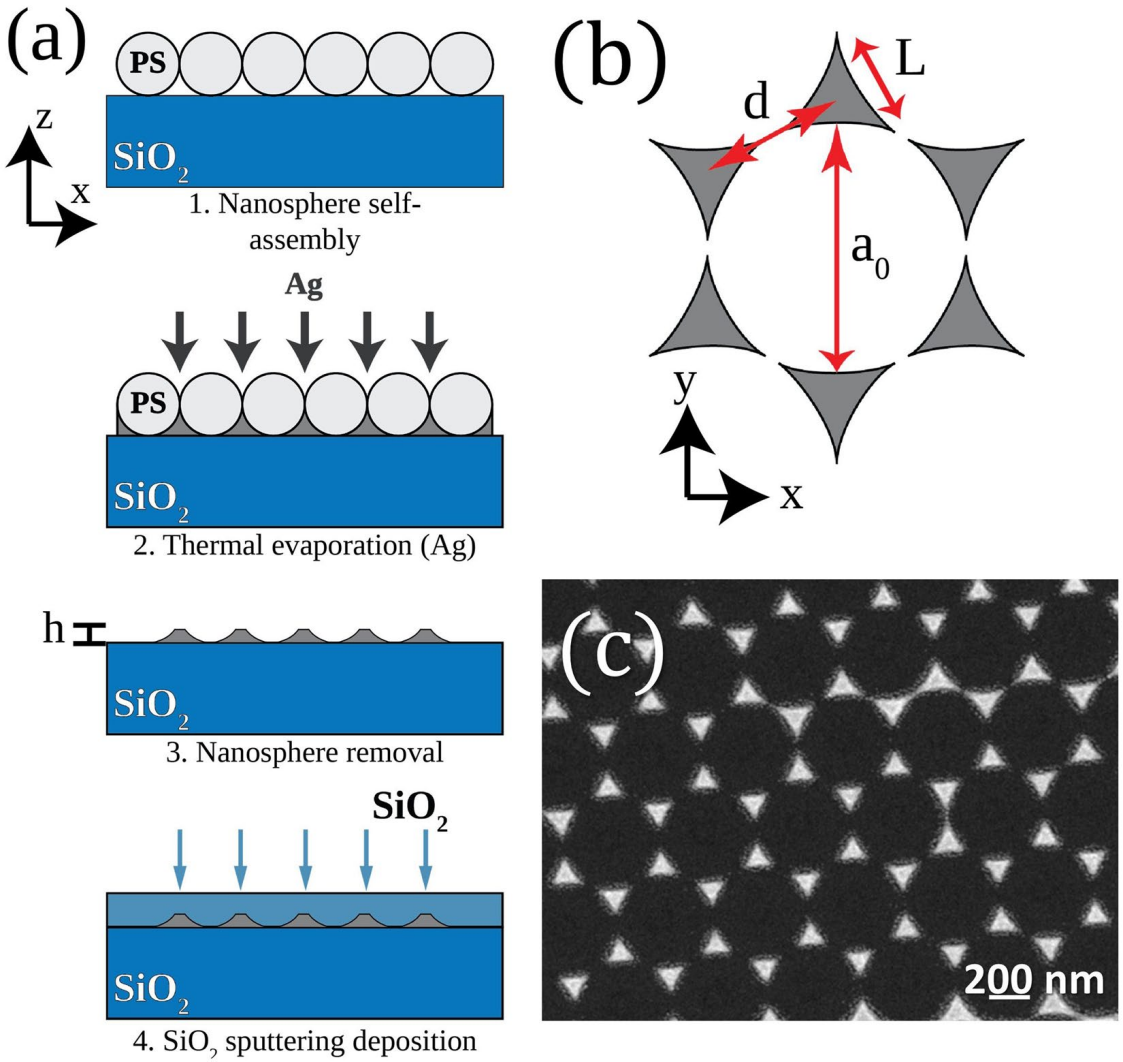
(**a**) MNPAs manufacturing process: (1) colloidal mask formation by self-assembly of PS nanospheres on a silica substrate; (2) thermal evaporation of Ag onto the PS nanospheres; (3) nanosphere removal; (4) deposition by magnetron sputtering of a protecting layer of silica. (**b**) Nanoprism array geometry. The definitions of the relevant geometric parameters of the nanoarray are: the lattice parameter is *a*_0_ (corresponding to the PS nanospheres diameter), the distance between the nanoprisms *d*, the nanoprisms length *L* and height *h*. (**c**) SEM image in plane view of the synthesized MNPAs.

The *nanosphere lithography* (NSL) technique was used in order to fabricate 2D ordered arrays of plasmonic Ag nanoprisms^12, 15–17^. The manufacturing process is briefly sketched in Fig. 1. First, polystyrene (PS) nanospheres were self-assembled on the surface of a clean silica glass substrate. The cleaning agent used was a “piranha” solution (H_2_SO_4_:H_2_O_2_, 3:1) while the PS nanospheres were of 522 nm nominal diameter (MicroParticles GmbH). Following the formation of the colloidal layer of nanospheres, thermal evaporation of Ag was performed. The deposited layer thickness was measured by the use of a calibrated quartz microbalance. Thereafter the polysterene nanospheres were mechanically removed by use of an adhesive tape. A silica layer was then deposited by magnetron sputtering on the MNPAs in order to prevent possible oxidation effects as well as physical damage. This top protective layer was estimated to be 160 nm thick.

### Structural and linear characterization

**Figure 2:**
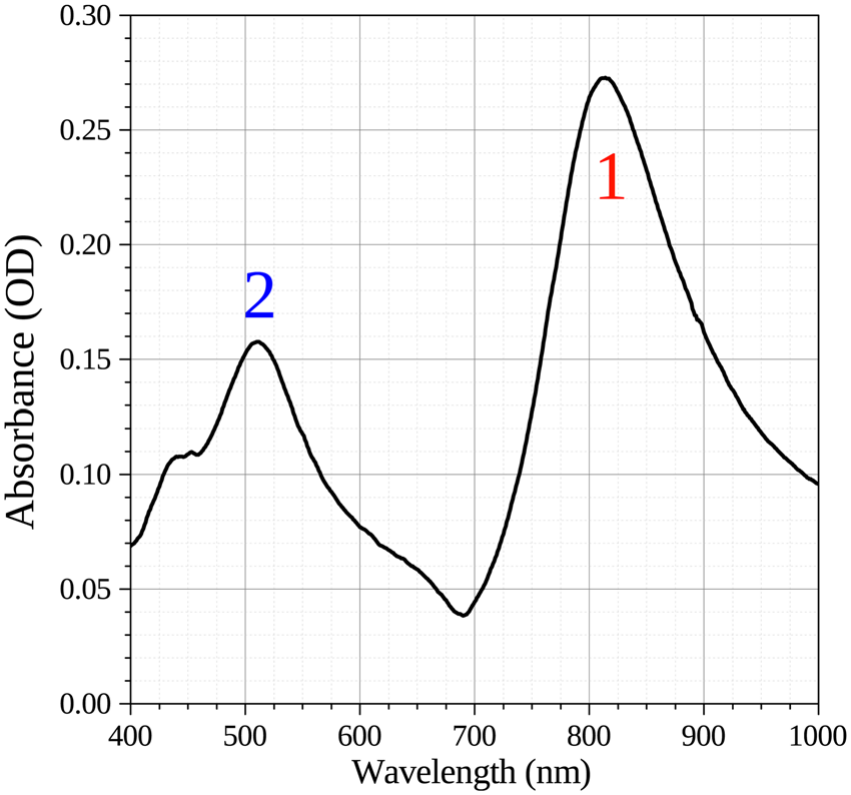
Optical absorbance spectrum of an Ag nanoprism array (height *h* = 70 nm), showing a characteristic LSPR response with well defined dipolar (1) and quadrupolar (2) peaks.

The resulting nanostructures were observed with a FE-SEM microscope (model LEO 1530, Zeiss) with a nominal operation voltage between 0.2 and 30 kV. The final height of the nanoprisms was measured by atomic force microscopy (AFM, model NT-MDT Nova Solver-PRO in non-contact mode). The relevant geometric parameters of the nanoarray are defined in label b in Fig. 1: *a*_0_ is the lattice parameter (corresponding to the PS nanospheres diameter), *d* is the distance between the nanoprisms, *L* the side length of each nanoprism and *h* their height. For the MNPAs investigated in the present work the parameters were *a*_0_ = 522 ± 5 nm, *d* = 302 ± 6 nm, and *L* = 155 ± 3 nm; concerning the nanoprisms height, two sets of samples were synthesized with *h* = 70 ± 2 nm, and *h* = 49 ± 2 nm. The linear absorption spectra of the nanoarrays, given by OD = −log(*I*/*I*_in_) with *I* the output intensity and *I*_in_ the input intensity, were recorded with a JASCO V670 dual beam spectrophotometer. The spectrum of the array of 70 nm height is shown in Fig. 2.

### FEM simulations

Finite Elements Method (FEM) electrodynamic simulations of the linear optical properties of the synthesized MNPAs were performed with the software COMSOL Multiphysics 5.1 by solving the Helmholtz equation in the frequency domain^18^. A hexagonal unit cell was considered to model the array of nanoprisms in the $\widehat{xy}$ plane. Each vertex of the hexagonal cell contains a third of a nanoprism and the tips of the nanoprisms were along the edges of the cell. In the direction of light propagation ($\hat{z}$ direction), the nanoprisms were placed on a silica substrate at the *z* = 0 plane and surrounded on top by a silica layer. In the $\hat{x}$ and $\hat{y}$ directions, periodic boundary conditions (PBC) were considered. Unphysical radiation backscattering from external boundaries was absorbed by using Perfectly Matched Layer (PML) sub-domains added over and below the simulated domains. To determine the silver dielectric functions to be used in the simulations, ellipsometry measurements were performed on planar Ag films made by thermal evaporation with the same conditions as for the MNPAs. A J. Woolham V-VASE spectroscopic ellipsometer was used for the measurements. The index of refraction of the silica substrate and top layer was set to *n* = 1.46. FE-SEM images of the synthesized samples were used to model the shape of the nanoprisms, while their height was obtained by AFM measurements.

### Nonlinear absorption measurements

The third order nonlinear absorption properties of the MNPAs were studied with the *z*-*scan* technique^14^, by performing *open aperture* (OA) measurements. The spectral dependence of the NLA was studied using a picosecond laser source coupled to an *optical parametric amplifier* (OPA). This allowed for a maximum pulse energy of up to 1 mJ as well as a tunable wavelength between 450 and 1100 nm, with pulses shorter than 20 ps and a repetition rate of 10 Hz. A 100 mm converging lens was used to focus the laser beam in the z-scan method. The beam waist *w*_0_ at the lens focus was measured using the razor-edge method^19^. At *λ*_0_ = 633 nm, *w*_0_ was about 30 *μ*m. Properly calibrated optical density filters were used to control the average peak intensity *I*_0_ at various points in the experiment. In order to prevent sample damage or modification during the scans, the laser beam peak intensity *I*_0_ was maintained under 1 GW/cm^2^ in all cases. Two Si photodetectors were used to detect both the transmitted intensity as well as the laser pulse energy as a reference to minimize the effects of laser fluctuations. For each point, the photodetectors response was measured with the help of an oscilloscope, and a 10% on peak integration window was chosen. The integrated value was then obtained by averaging over 250 pulses for each z position.

## Results and Discussion

The optical response of plasmonic nanostructures depends on several variables such as the size, shape, composition of the nanoparticles, the dielectric contrast with the host medium and the coupling effects amongst the nanostructures, which is in turn dependent on their arrangement^20–22^. As an example, in Fig. 2 we report the optical absorbance spectrum of a 70 nm height Ag nanoprism array embedded in silica. This spectrum clearly shows two resonance peaks at 811 and 511 nm; these peaks call on the different LSPR characteristics of the nanoarrays with the peak at 811 nm (label 1 in Fig. 2) being the result of the dipolar resonance while the on at 511 nm (label 2 in Fig. 2) due to a quadrupolar response^13, 23–25^. The small shoulder visible in the spectrum at around 450 is instead very likely related to the presence of defects in the experimental sample.

**Figure 3:**
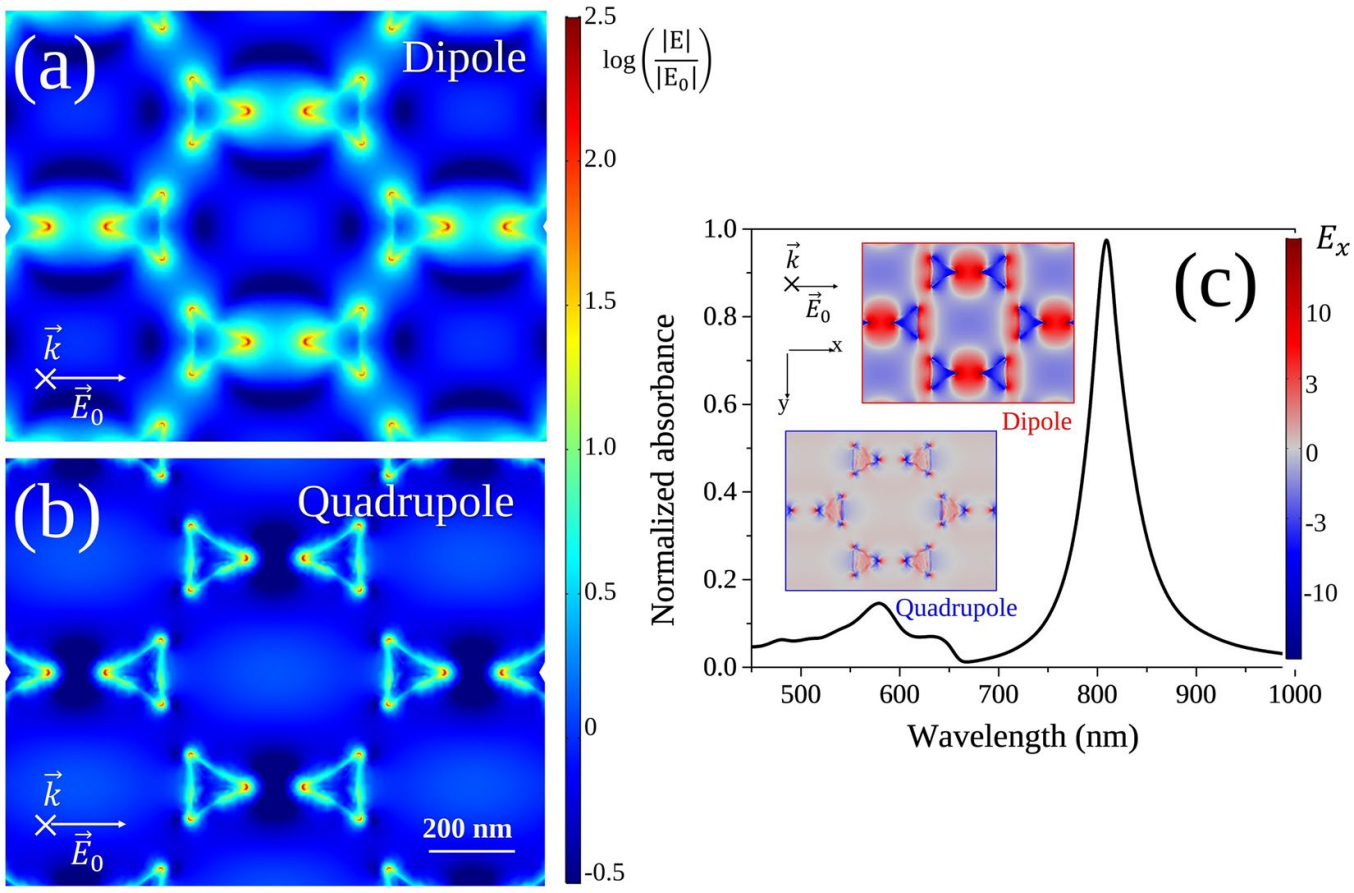
Simulated maps of local-electric field enhancement factor, in logarithmic scale, evaluated at the dipolar (**a**) and quadrupolar (**b**) SPR resonance peaks. *E*_0_ is the incident electric field amplitude. Panel (**c**) portrays the normalized absorbance spectrum obtained by FEM Simulations. The insets show the maps of the $\hat{x}$ component of the electric field *E*_*x*_ (i.e., parallel to the incident field *E*_0_) evaluated at the dipolar and quadrupolar resonances.

Numerical analysis of the local field enhancement due to the nanostructure has suggested that the LSPR peaks are mostly determined by the interaction found in the dielectric-metal interface^13^. Therefore, since the addition of the silica protective cover greatly increases the surface area between the nanoparticles and dielectric, the LSPR response is expected to be reinforced in our samples. Finite elements method (FEM) simulations of the local-electric field in the investigated nanoprism arrays have been performed at both the dipolar and quadrupolar resonance wavelengths. The corresponding simulated maps of local-field enhancement factor *F* = |*E*|/*E*_0_ are reported in Fig. 3. In Fig. 3(a,b) we reported the simulated absorbance spectrum (normalized to its maximum). The insets show the maps of the $\hat{x}$ component of the electric field, *E*_*x*_ (i.e., parallel to the incident field *E*_0_) evaluated at the two resonances. In the simulations, it can be seen that the electric field distribution is greatly confined at the tips of the nanoprism elements and the confinement is stronger at the quadrupolar resonance due to the shorter decay length of the multipolar plasmon mode. Moreover a total reinforcement of the electric field magnitude of more than 300 times (log(F) = 2.5) is obtained due to near-field coupling among the nanoprisms in the array. In this work, we focused our efforts on two different aspects of the nonlinear response of MNPAs. In first instance, we studied the irradiance dependence of the NLA at resonance with both the dipolar and quadrupolar absorption feature. This is in order to understand the nature of the nonlinearity. In a second part, we studied the spectral dependence of the response across the dipolar plasmon resonance, to look for spectral regimes with negligible nonlinear absorption. The measurements were done with a peak intensity *I*_0_ of up to 500 MW/cm^2^, a relatively low value in comparison to similar experiments^13, 26, 27^. Because of the signal-to-noise ratio of our experimental set-up, no measurement at intensities lower than *I*_0_ = 20 MW/cm^2^ showed useful results. These experimental conditions avoided damage or permanent modification in the sample as it was scanned, as checked by issuing back and forth scans with the same intensities.

### Irradiance dependence

**Figure 4:**
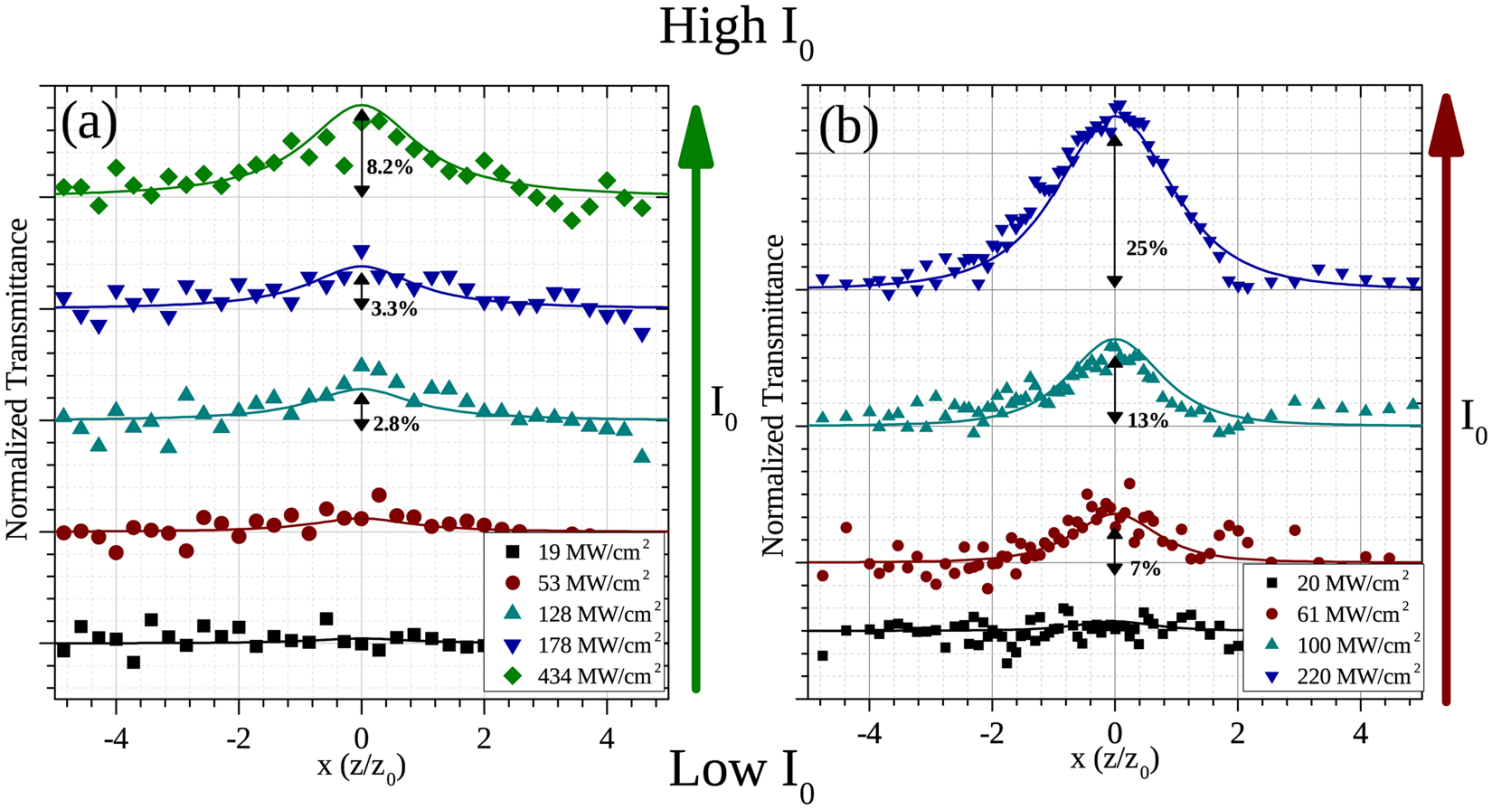
Open-aperture (OA) z-scans of Ag nanoprism array of 70 nm height, measured at (**a**) the quadrupolar resonance (*λ*_*Q*_ = 511 nm) and (**b**) the dipolar resonance (*λ*_*D*_ = 811 nm). The scans were taken with different laser peak intensities *I*_0_, as indicated in the legend. Solid lines are best-fits to the experimental data.

Figure 4 shows the open aperture z-scan results obtained for both the quadrupolar and dipolar resonances taken at different input irradiance values. The results show in all cases the presence of saturable absorption, i.e. a transmittance that increases with irradiance, resulting in a maximum at the focal plane (*z* = 0). This was expected due to the wavelength being on resonance for both cases. For the quadrupolar response at *λ*_*Q*_ = 511 nm (Fig. 4a) however, the magnitude of the NLA effect with the input pulse intensity is considerably smaller than that at the dipolar resonance at *λ*_*D*_ = 811 nm for similar input irradiance values. For the study at 811 nm, as shown in Fig. 4b, the effect also increases monotonically when the input irradiance is augmented but seems to start saturating at input irradiances around 200 MW/cm^2^.

**Figure 5:**
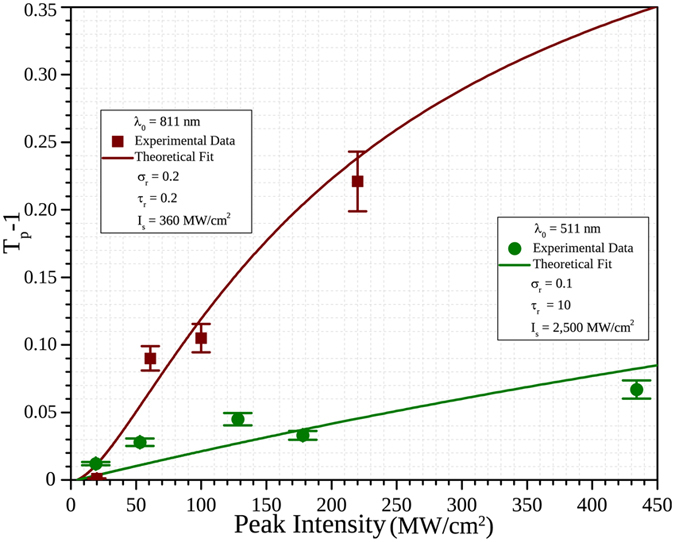
Experimental data and best-fit calculations for the maximum NLA change given by *T*_*p*_ − 1 at both the dipolar (red squares for experiment data and red solid line for theoretical fit) and the quadrupolar (green circles for experiment data and green solid line for theoretical fit) regimes. While the quadrupolar regime shows an early saturation effect at low intensities, the dipolar response saturates at much higher peak intensities.

A diminishing or increasing transmittance is usually modeled by an irradiance dependent absorption coefficient *α*(*I*) given by *α*(*I*) = *α*_0_ + *βI*, where *β* is the so called *nonlinear absorption coefficient*; where *β* can be positive (*induced* or *reverse saturable absorption*, RSA), or negative (*saturable absorption*, SA). This model implies however that the maximum transmittance increases or decreases linearly with irradiance, something that is often not observed in the experiments. In Fig. 5, we show the value of the maximum transmittance change *T*_*p*_ − 1, observed for the z-scan results in Fig. 4, as a function of the input irradiance. For both the dipolar (811 nm) and the quadrupolar (511 nm) resonances, it is easily seen that the transmittance change does not increase linearly with the input irradiance, but rather tends to saturate at higher irradiances. This saturation is sometimes taken into account by modifying the expression for *α*(*I*) given by equation 1 to *α*(*I*) = *α*_0_ + *β*_0_ *I*(1 + *I*/*I*_*s*_)^13^, where a saturation irradiance *I*_*s*_ is introduced and *β*_0_ is now the *unsaturated* or *low irradiance nonlinear absorption coefficient*, which again could be positive as well as negative to account for induced or saturable absorption respectively. On the other hand, for resonant interactions, a two-level model results in *α*(*I*) = *α*_0_/(1 + *I*/*I*_*s*_), which represents a saturable absorption with an irradiance dependence that is clearly not linear, but saturates at higher irradiances. Moreover, when there are higher lying energy states that can be accessible from the first excited state by further light absorption, a three-level system needs to be employed. Such a three-level has been used before to explain the spectral and irradiance dependence of the nonlinear absorption of different materials^28, 29^, and can produce a NLA that changes sign at different input irradiances, which has been observed. For the MNPAs systems presented here, the presence of the quadrupolar plasmon absorption indicates that further absorption beyond the excitation of the dipolar plasmon is possible, and therefore this model should be used. In the most general form of this model, transitions between the three levels are allowed: direct transitions from the ground state to either of the excited states |2〉 or |3〉, and excited state absorption, i.e. |2〉 → |3〉 transitions. Decay from level 3 into either level |1〉^28^, or level |2〉^29^ has been considered, with no change in the structure of the irradiance dependence of the nonlinearity obtained. The resulting rate equations are then solved in the steady state, and an effective susceptibility ${\chi }_{eff}^{\mathrm{(3)}}$, can be calculated from the solution. In the present case, we will consider only the direct transition from the ground state and the excited state absorption, i.e. |1〉 → |2〉 and |2〉 → |3〉 transitions and hence the absorption cross-section ${\sigma }_{13}\cong 0$. For simplicity, we will also consider decay from level |3〉 only through the |3〉 → |2〉 transition, but again, this is not a very restrictive assumption.

For the case of NLA, the intensity dependent absorption coefficient can be extracted from ${\rm{Im}}\{{\chi }_{eff}^{\mathrm{(3)}}\}$ to yield^29^:

1 $$\alpha (I)={\alpha }_{0}\{\frac{1+{\sigma }_{r}(I/{I}_{s}\mathrm{)(1}+{\tau }_{r})}{1+(I/{I}_{s}\mathrm{)(2}+{\sigma }_{r}{\tau }_{r})+\mathrm{3(}I/{I}_{s}{)}^{2}{\sigma }_{r}{\tau }_{r}}\},$$

where *α*_0_ is the linear absorption coefficient (*α*_0_ = *σ*_12_ *N*_0_ with *N*_0_ the number density of the material), ${\sigma }_{r}\equiv {\sigma }_{23}/{\sigma }_{21}$, ${\tau }_{r}\equiv {\tau }_{32}/{\tau }_{21}$ and *σ*_*ij*_ and *τ*_*ij*_ are the absorption cross-section and relaxations times for the *i* → *j* transitions, respectively. For the purely absorptive nonlinear case, expression (1) is used to describe propagation through the sample by calculating:

2 $$dI/dz^{\prime} =-\alpha (I)/I,$$

from *z*′ = 0 to *z*′ = *L*, with *L* the sample thickness. To obtain the open aperture z-scan trace, this integral must be calculated at each *z* position of the sample as well as for each *I*_0_, as the position dependent irradiance *I*(*z*) changes with *z*. The irradiance dependence of the open aperture z-scan data was fitted with this model for both the dipolar (*λ*_*D*_ = 811 nm) and quadrupolar (*λ*_*Q*_ = 511 nm) resonances. In Figs 4 and 5, the fits made with this model for each data set at a given input irradiance and the fitted irradiance dependence for *T*_*p*_ − 1 at 511 and 811 nm are shown. These fits were obtained via numerical calculation of the solution for equation 1 at each z position. The model fits very well the irradiance dependence in both cases and the fitting parameters *τ*_*r*_, *σ*_*r*_ and *I*_*s*_ for each wavelength were obtained and are presented in Table 1.

**Table 1:** Fitting parameters for the data at the two resonances.

*λ*_0_ (nm)	*σ*_*r*_	*τ*_*r*_	I_*s*_ (MW/cm^2^)
511	0.2	0.2	360
811	0.1	10	2500

One advantage of this model and calculation method is that only one set of parameters per wavelength is needed to obtain traces for any given z-scan set without losing physical relevance. Although at first sight there are too many free parameters for a unique fit, fitting the data at different input irradiances eliminates any ambiguities in the determination of the model parameters. The fitting to the model allows us in first instance to extrapolate the results to higher irradiances than those employed. Furthermore, the parameters extracted from the fit, together with linear optical measurements (which allow determination of *σ*_12_ and *τ*_21_) can be used to obtain *σ*_23_ and *τ*_32_, which are usually experimentally difficult to determine. Closed-aperture z-scans will in principle present also such saturation effects. However, not having a very good spatial shape for the pulses precluded us from making any quantitative studies.

The large nonlinear response observed in a sample that is only 70 nm thick can be seen as the result of the field enhancement observed in the numerical calculations, and of the coherent addition of the contribution of each nanoprism element. The observed nonlinear absorption is markedly larger for the dipolar resonance as compared to the quadrupolar. This is expected since the absorption peak is stronger for the former, indicating a larger dipolar moment. The nonlinear response will be also a fraction of this moment, giving rise to the larger response.

### Wavelength dependence

Much interest has been placed in studying the “on peak” nonlinear response of plasmonic materials, yet its fine wavelength dependence is very rarely explored. This is due to the assumption that the nonlinear response is maximum strictly on resonance with the LSPR, an assumption that is usually not tested^30^. This, altogether with the nonlinear plasmonic nanomaterials potential for frequency based multiplexing applications, makes it clear that the fine wavelength dependence of plasmonic materials is of special interest.

**Figure 6:**
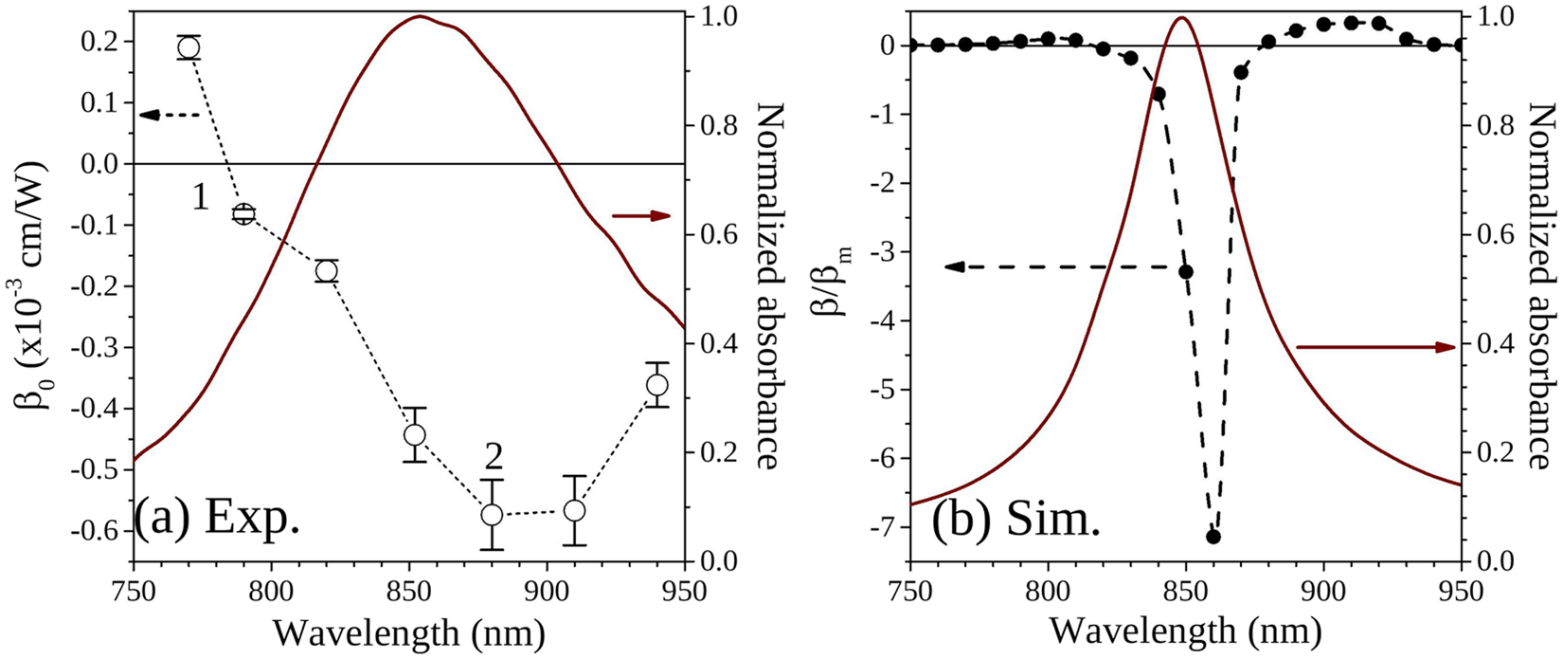
Wavelength dependence around the dipolar resonance peak of NLA of MNPAs with a height h = 49 nm. Panel (**a**) shows the unsaturated NLA coefficient *β*_0_ estimated from the open-aperture z-scan experimental data. Also shown is the normalized linear absorbance spectrum. Two regions of interest are noted, with label 1 showing a change of sign in the nonlinearity and thus a cross by zero, and label 2 presenting a red-shift in the maximum nonlinear response with respect to the linear resonance. Panel (**b**) shows the results calculated with equation (4); the normalized linear absorbance spectrum obtained from FEM simulations is also reported.

To this aim, in the present work we studied the spectral dependence of the nonlinear absorption properties of the investigated silver nanoprism arrays around the dipolar resonance. In order to avoid possible damage to the sample, a new one containing nanoprism arrays of height *h* = 49 ± 2 nm (and same lattice parameter *a*_0_ = 522 ± 5 nm) was synthesized, whose dipolar LSPR is thus shifted at a slightly longer wavelength of 850 nm. Moreover, in this way the whole peak was within the optical working range of the OPA (i.e. above the signal-to-idler crossing point at 710 nm). We concentrated the study of wavelength around the dipolar resonance only, since the nonlinear response is considerably larger than that of the quadrupolar one. All the measurements were made at the same irradiance of about *I*_0_ = 120 MW/cm^2^, in order to establish a direct comparison between the data at different *λ*. This relatively low irradiance *I*_0_ value was chosen in order to minimize possible saturation effects, and to be as close as possible to a purely third order nonlinearity. In the limit of low irradiance, the three-level model expression given by equation 1 for *α*(*I*) can be approximated to yield $\alpha (I)\simeq {\alpha }_{0}+{\beta }_{0}I$, where *β*_0_ = *α*_0_(*σ*_*r*_ − 2)/*I*_*s*_ is the unsaturated NLA coefficient. The figure *β*_0_ can take positive (corresponding to RSA) or negative (SA) values. Therefore, the full three-level model was used to fit the open aperture z-scan data, and the values of *β*_0_ at the used wavelengths were calculated in this way. Figure 6a shows the calculated *β*_0_ values together with the linear absorbance spectrum of the sample, normalized to its maximum. For wavelengths shorter than 770 nm (label 1 in Fig. 6a) the results show a positive value of *β*_0_, indicating an induced absorption process or RSA. In addition, for wavelengths longer than 790 nm, the results show the presence of saturable absorption, characterized by a negative *β*_0_. Another thing that can be observed from the results is that the largest |*β*_0_| value measured is not at the resonant peak, *λ*_*D*_ = 850 nm, but at a rather longer wavelength of 880 nm. The fact that the maximum |*β*_0_| value does not correspond with the absorption peak, as would be expected for a single resonance, has probably to do with the presence of the shorter wavelength quadrupole resonance, and its influence on the NLA spectral dependence^29^.

Moreover, it has to be noted that for nanostructures that present high curvature regions, as the tips of the nanoprisms in the present case, the spectral position of the maximum field enhancement may be different according to the region considered, e.g. the hot-spot regions at the nanoprisms tips or the whole nanoprism^31^. Since the formation of these hot-spots in the MNPAs control their nonlinear behavior, whereas their linear absorbance depends on the average electric field in the nanoarray, this may account for spectral differences in the maximum linear and nonlinear response of these nanosystems. In order to further investigate this aspect, we performed FEM electrodynamic simulations. Indeed, the spectral dependence observed in the nonlinear absorption response of the investigated MNPAs can be explained as a consequence of the wavelength-dependent distribution of the local electric field in the nanoprism array. Particularly, the electric field distribution calculated by FEM simulations can be used to estimate the effective third-order nonlinear susceptibility, ${\chi }_{eff}^{\mathrm{(3)}}$, of the investigated nanosystems. Indeed, following the approach used by Debrus *et al*.^32, 33^, for metal-dielectric composite materials ${\chi }_{eff}^{\mathrm{(3)}}$ can be expressed in the form:

3 $${\chi }_{{eff}}^{\mathrm{(3)}}={\chi }_{m}^{\mathrm{(3)}}\frac{1}{V}\frac{\int {E}^{2}(\lambda ){|E(\lambda )|}^{2}dV}{{E}_{0}^{2}{|{E}_{0}|}^{2}V},$$

where V is the unit cell volume of the composite material, *E*_0_ is the incident electric field amplitude and *E* is the local-field amplitude in the material. From expression (3), assuming that the nonlinear contribution of the silica matrix is negligible with respect to that of the metal and that the complex cubic susceptibility of the metal component, ${\chi }_{m}^{\mathrm{(3)}}$, is dominated by its imaginary part, the nonlinear absorption coefficient of the MNPAs investigated in the present work can be expressed according to the equation^34, 35^:

4 $$\frac{\beta (\lambda )}{{\beta }_{m}(\lambda )}={\rm{Re}}\{\frac{\int {E}^{2}(\lambda ){|E(\lambda )|}^{2}dV}{{E}_{0}^{2}{|{E}_{0}|}^{2}V}\}\frac{{n}_{0}{(\lambda )}^{2}}{{n}_{{\rm{eff}}}{(\lambda )}^{2}},$$

where *n*_0_ is the refractive index, *n*_eff_ is the effective refractive index of the MNPA and *β*_*m*_ is the intrinsic nonlinear absorption coefficient of a homogeneous silver film. Very recently, this approach was also successfully used by our group to describe the polarization-dependent nonlinear absorption properties of similar silver nanoprism arrays^35^. The value of *n*_0_ at the different wavelengths was experimentally determined by spectroscopic ellipsometry measurements performed on a silver film made by thermal evaporation with the same conditions used for the synthesis of the MNPAs. *n*_eff_(*λ*) was instead computed with the S-matrix method^36^ by using the local electric field distribution at the different *λ* obtained by FEM simulations. Moreover, for a homogenous metallic film, far from the interband transitions as in the present case, *β*_*m*_ is expected to have a very weak wavelength dependence^34^. Thus, the main spectral features in the nonlinear response of the investigated MNPAs can be accounted by evaluating the right-hand term in equation 4. In Fig. 6b we reported the results of this calculation in the wavelength range around the dipolar resonance used in the experiment (black dots, left-hand scale). The simulated absorbance spectrum of the samples (normalized to its maximum) is also reported (red curve, right-hand scale). The calculated LSPR absorbance peak is narrower than the experimental one, and this is probably due to the presence of defects in the fabricated nanostructures, that broaden the absorption band in the experimental samples. Nonetheless, a very good agreement between simulated and experimental results was reached. Particularly, the simulations show that the maximum nonlinear absorption response is observed at a wavelength slightly longer than the resonance peak, in agreement with the experimental results. Moreover, a change of sign from SA on resonance to RSA for shorter and longer wavelengths was demonstrated. Experimentally, such a change of sign was observed for wavelengths shorter than the resonance (Fig. 6a), while above the resonance it is still expected, but beyond the wavelength range accessible for the experiment, so it could not be observed. Anyhow, it is worth noticing that both the simulation and experimental results highlighted the existence of two spectral regions where NLA changes from saturable to induced absorption. Therefore, the NLA effect is expected to become negligible or very small at these wavelengths. Usually nonlinear absorption constitutes a deleterious effect for applications depending on nonlinear refraction, such as all-optical switching devices, where more than one device can be used in cascade^37^. A regime where NLA is negligible is therefore very interesting for possible applications in all-optical switching, and it is found for wavelengths around 780 nm. This probably happens for wavelengths larger than 950 nm, but due to our detection system limitations, we could not prove it in our experiments.

## Conclusions

The nonlinear absorption properties of ordered metallic Ag nanoprism arrays embedded in silica and their irradiance and wavelength dependence have been studied by OA z-scan measurements with a wavelength-tunable ps laser beam. By setting the laser wavelength to the dipolar and quadrupolar plasmon resonances of the nanoarray, the gamut of nonlinear absorption phenomena has been observed and measured. The results show a remarkable reinforcement of the nonlinear absorption response at low intensities due to the great magnitude of the LSPR local field reinforcement. The quadrupolar and dipolar NLA responses are quite different and offer a great degree of freedom when designing devices based on effects such as SA and RSA. One of the most interesting results in this regard is the cancellation of the nonlinear response near the LSPR dipolar peak. This has been both observed experimentally and theoretically demonstrated by FEM electrodynamic simulations. Moreover, our results suggest that great consideration should be placed when deciding the manufacturing parameters of plasmonic materials for applications due to less than obvious wavelength-dependent nonlinear absorption effects, e.g. for maximum nonlinear response. The improved response of the investigated silver nanoprism arrays, along with their wide gamut of nonlinear absorption characteristics, makes these nanosystems extremely attractive for photonic and plasmonic ultrafast devices.
